# Latent brain state dynamics distinguish behavioral variability, impaired decision-making, and inattention

**DOI:** 10.1038/s41380-021-01022-3

**Published:** 2021-02-15

**Authors:** Weidong Cai, Stacie L. Warren, Katherine Duberg, Bruce Pennington, Stephen P. Hinshaw, Vinod Menon

**Affiliations:** 1 Department of Psychiatry & Behavioral Sciences, Stanford University School of Medicine, Stanford, CA USA; 2 Wu Tsai Neuroscience Institute, Stanford University, Stanford, CA USA; 3 Department of Psychology, Palo Alto University, Palo Alto, CA USA; 4 Department of Psychology, University of Denver, Denver, CO USA; 5 Department of Psychology, University of California, Berkeley, CA USA; 6 Department of Psychiatry & Behavioral Sciences, University of California, San Francisco, CA USA; 7 Department of Neurology & Neurological Sciences, Stanford University School of Medicine, Stanford, CA USA

**Keywords:** Neuroscience, ADHD

## Abstract

Children with Attention Deficit Hyperactivity Disorder (ADHD) have prominent deficits in sustained attention that manifest as elevated intra-individual response variability and poor decision-making. Influential neurocognitive models have linked attentional fluctuations to aberrant brain dynamics, but these models have not been tested with computationally rigorous procedures. Here we use a Research Domain Criteria approach, drift-diffusion modeling of behavior, and a novel Bayesian Switching Dynamic System unsupervised learning algorithm, with ultrafast temporal resolution (490 ms) whole-brain task-fMRI data, to investigate latent brain state dynamics of salience, frontoparietal, and default mode networks and their relation to response variability, latent decision-making processes, and inattention. Our analyses revealed that occurrence of a task-optimal latent brain state predicted decreased intra-individual response variability and increased evidence accumulation related to decision-making. In contrast, occurrence and dwell time of a non-optimal latent brain state predicted inattention symptoms and furthermore, in a categorical analysis, distinguished children with ADHD from controls. Importantly, functional connectivity between salience and frontoparietal networks predicted rate of evidence accumulation to a decision threshold, whereas functional connectivity between salience and default mode networks predicted inattention. Taken together, our computational modeling reveals dissociable latent brain state features underlying response variability, impaired decision-making, and inattentional symptoms common to ADHD. Our findings provide novel insights into the neurobiology of attention deficits in children.

## Introduction

Attention Deficit Hyperactivity Disorder (ADHD) is a highly prevalent neurodevelopmental disorder affecting 5–10% of children worldwide [1], and is characterized by heterogeneous behaviors, symptoms, and developmental trajectories. Isolating phenotypic dimensions of attentional symptoms has the potential to resolve heterogeneity, advancing nosology, and clinical practice. A consistent behavioral phenotype associated with attentional deficits is intra-individual response variability (IIRV), which measures trial-to-trial response variance [2, 3]. Increased IIRV in ADHD is associated with poor sustained attention and problems with cognitive control [4, 5]. Although the etiology of IIRV is not known, some have proposed that abnormal fluctuations of latent brain states underlying the occurrence of task-irrelevant cognition is associated with unstable performance [6, 7]. However, this hypothesis has never been rigorously tested with appropriate neurocognitive and computational models. We used a novel Bayesian unsupervised learning model and a Research Domain Criteria (RDoC) approach [8] to uncover dynamics of latent brain states during cognitive task performance and its relation to behavioral variability and attention problems in children. Consistent with the objectives of RDoC, we examined IIRV, fluctuations in attention, and decision-making processes as continuous distributions, integrating cognitive and neurobiological systems to advance our understanding of attention symptom dimensions associated with ADHD [9].

A large body of human functional neuroimaging studies has uncovered a core set of distributed regions in the salience network (SN), anchored in the anterior insula (AI) and dorsal anterior cingulate cortex, and frontal-parietal network (FPN), anchored in the dorsolateral prefrontal and posterior parietal cortex, that support attention and cognitive control [10–17]. Multiple meta-analyses have confirmed consistent engagement of SN and FPN nodes across attentionally demanding tasks [10, 18–22]. Growing evidence indicates that the default mode network (DMN), anchored in the posterior cingulate cortex (PCC) and ventromedial prefrontal cortex (VMPFC), influences attention and cognitive control [23–26].

Meta-analyses of fMRI studies in children with ADHD have reported abnormal activation in prefrontal and parietal brain areas associated with the SN, FPN, and DMN during a variety of cognitive control tasks [27–30]. Aberrant intrinsic network interaction among the SN, FPN, and DMN and weak task-modulated connectivity between SN and FPN regions are associated with poorer attentional performance and more severe inattention symptoms in ADHD [6, 31]. Previous studies using resting-state fMRI have reported DMN abnormalities in individuals with ADHD [32–34]. However, little is known about brain state dynamics associated with these networks during cognitive performance and their contributions to attention problems. Increased IIRV, a robust behavioral phenomenon associated with ADHD, has been related to attention problems [35, 36], and attention is a critical element influencing decision-making processes [37]. Common biological processes likely contribute centrally to these interrelated cognitive processes and behavioral deficits, but the specific brain mechanisms remain unknown. A rigorous quantitative approach to behavioral analysis and systems neuroscience models of brain state dynamics are needed to better characterize the association between overt IIRV, latent decision-making processes underlying IIRV, and attentional variability across children.

We used a novel Bayesian switching linear dynamic systems (BSDS) model [38] to probe fluctuations of latent brain states and their relation to behavior. Investigating time-varying, context-dependent brain states is a non-trivial computational problem because of the inherent complexities of nonlinear and latent dynamical process that characterize brain function [38–41]. BSDS addresses this problem by implementing an unsupervised Bayesian learning algorithm that determines hidden (latent) brain states and dynamic state transitions automatically from observed data. Briefly, each brain state is associated with a unique dynamical process that captures time-varying activation and functional connectivity in an optimal latent subspace. Furthermore, BSDS applies a hidden Markov model to latent space variables of the observed data, resulting in a parsimonious model of generators underlying the observed data. Importantly, BSDS does not require arbitrary moving windows nor does it impose temporal boundaries associated with predefined task conditions, which are major limitations of existing methods for probing dynamic processes in the human brain [42].

**Fig. 1:**
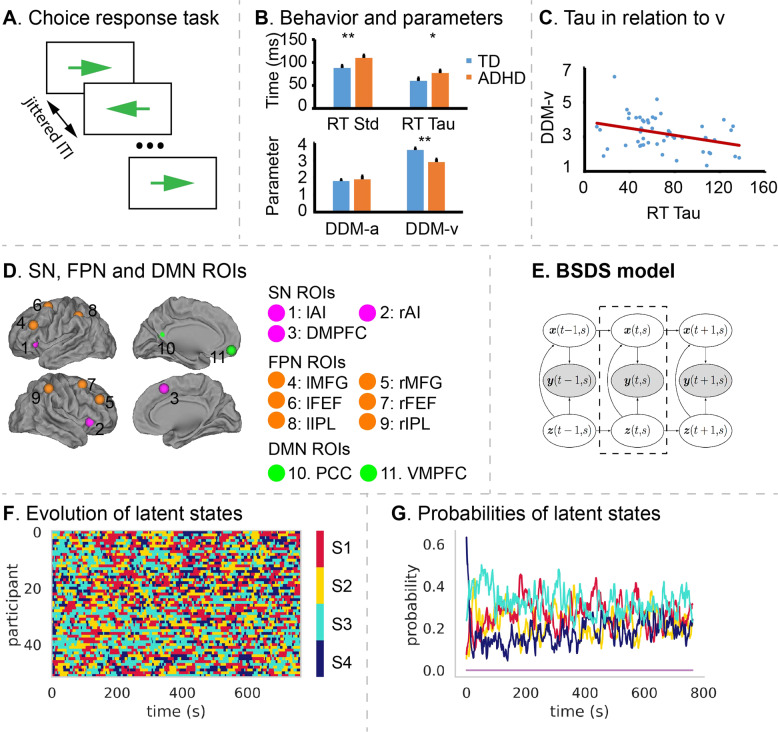
Task paradigm, behavior and latent brain states. **A** Illustration of the choice response event-related fMRI task. **B** Children with ADHD have significantly larger RT standard deviation (Std) and tau than TD children, whereas TD children have significantly higher information accumulation speed (v) than children with ADHD. **C** RT tau is negatively correlated with information accumulation speed (v) (*r* = −0.34, *p* = 0.01). **D** Regions of interest (ROIs) include key nodes in the salience (SN), frontal-parietal (FPN) and default mode networks (DMN). **E** Schematic illustration of the BSDS model. **F** Temporal evolution of latent brain states in the choice response task. Each row represents one subject, each column represent one data point (fMRI volume). **G** Dynamic changes in posterior probability of latent brain states during the choice response task (averaged across participants).

IIRV has been examined through a variety of cognitive paradigms involving multiple levels of task difficulty through manipulation of attention and cognitive control demands. Participants adjust their response strategies proactively based on the anticipation of upcoming stimuli [43, 44] or feedback from a previous response outcome [45], leading to fluctuating RTs and elevated response variability. Therefore, IIRV is dependent on task complexity [5] and increased IIRV is driven by attentional fluctuation or trial-by-trial behavioral adaptation. To minimize trial-by-trial behavioral adaptation induced by infrequent stimuli and task difficulty, we studied response variability in children using a simple choice response task. The simple choice response task requires participants to make left or right button presses in response to left-pointing or right-pointing arrows, respectively (Fig. 1A). As the behavioral and attentional symptoms associated with ADHD exist as continuous distributions in the general population, we studied IIRV across typically developing (TD) children and children with ADHD.

IIRV is often indexed using standard deviation (std) from the Gaussian model [46]. However, RT distributions are typically skewed distributions, which fit better with an ex-Gaussian model than a Gaussian model [47, 48]. The ex-Gaussian model includes three parameters: mu, sigma, and tau. Mu and sigma are the mean and std of the Gaussian component, whereas tau represents the exponential component, which captures the tail of the skewed distribution. Previous studies have shown that std and tau can differentiate children with ADHD from TD children [2, 46, 49, 50]. The present study used both std and tau to further quantitatively characterize IIRV in children. We predicted that std and tau would correlate with core inattention symptoms and, additionally that, in categorical analyses, children with ADHD would have larger std and tau than TD children.

Although IIRV is a widely used measure of behavioral instability that indexes fluctuations in attention, it does not capture the underlying decision-making components of such variability. To address this, we examined latent cognitive processes underlying behavioral variability by modeling decision-making processes using a hierarchical drift-diffusion model (HDDM) [51]. HDDM estimates three latent components associated with decision-making: decision threshold or response caution, drift rate or how fast evidence is accumulated to reach the threshold, and non-decision time, or the perceptual processes engaged prior to the onset of evidence accumulation [52]. Models of attention suggest that attention has a strong impact on the rate at which evidence accumulates in the decision-making stage, indexed by the drift rate [37]. We predicted that impulsivity would be associated with a smaller decision threshold reflecting lower response caution, whereas deficits in sustained attention would impact drift rate reflecting reduced and noisier evidence accumulation.

Notably, we used BSDS with ultrafast resolution fMRI (temporal resolution = 490 ms) to determine latent brain states during performance of the choice response task and to determine the relation between its dynamic temporal properties and three distinct measures of behavior: (1) response variability, (2) latent measures of decision-making derived using HDDM, and (3) clinical phenotypic measures associated with inattention. Specifically, we examined whether engagement of a task-optimal dynamic brain state would reduce behavioral variability and improve decision-making processes during cognitive performance. We then examined how behavioral variability, decision-making, and inattention are related to functional connectivity between SN, FPN, and DMN. Finally, as a follow-up to our dimensional analyses, we examined whether behavior and brain state dynamics could accurately distinguish children with ADHD from TD children.

## Results

### Demographic profile

Fifty-two subjects, including 29 children with a clinical diagnosis of ADHD (11 female/18 male) and 23 TD children (11 female/12 male), completed the study. The two groups did not differ in age, gender, or head motion during task-fMRI scanning (all *p*s > 0.2, two-sample *t*-test, Table S1). Children with ADHD had significantly higher inattention and hyperactivity/impulsivity scores than TD children (all *p*s < 0.001, two-sample *t*-test, Table S1).

### Behavioral performance: dimensional analysis

Table S1 summarizes behavioral performance. Participants completed the in-scanner simple choice response task with high accuracies (95 ± 4%) and fast reaction time (RT) (477 ± 56 ms), suggesting good overall performance.

Dimensional analyses revealed that accuracy negatively correlated with inattention scores (*r* = −0.31, *p* = 0.02) but not with hyperactivity/impulsivity scores (*p* > 0.29). RT was not significantly correlated with either attentional score (*p* > 0.1). Results suggest that inattention, but not hyperactivity/impulsivity, is associated with behavioral performance in the choice response task.

### Behavioral performance: categorical analysis

Categorical analyses revealed a significant between-group difference in task accuracy (*t* = 3.08, *p* = 0.004, two-sample *t*-test) but not in average RT (*p*s > 0.2), suggesting that children with ADHD have relatively poorer performance compared to TD children.

### Behavioral variability: dimensional analysis

We then examined IIRV using RT std, sigma, and tau.

Dimensional analyses revealed that RT std was marginally correlated with inattention scores (*r* = 0.27, *p* = 0.05), but not with hyperactivity/impulsivity scores (*p* > 0.05). RT sigma was not significantly correlated with either score (*p* > 0.7), and RT tau was marginally correlated with inattention scores (*r* = 0.25, *p* = 0.06), but not with hyperactivity/impulsivity scores (*p* > 0.1). These results demonstrate that covariance between behavioral variability and inattention is driven by the tail of the ex-Gaussian distribution of RT, indexed by tau, rather than variability in the normal distribution component.

### Behavioral variability: categorical analysis

Categorical analyses revealed significant differences in RT std (*t* = 2.57, *p* = 0.01, two-sample *t*-test, Fig. 1B) and RT tau (*t* = 2, *p* = 0.05, two-sample *t*-test, Fig. 1B), but not RT sigma (*p* = 0.2), indicating that children with ADHD have less stable behavioral performance than TD children.

### Latent decision-making processes: dimensional analysis

Next, we used HDDM to fit a drift-diffusion process to each child’s performance and estimated the decision boundary (*a*), drift rate (*v*) and non-decision time (*t*). Drift rate (*v*) was significantly correlated with tau from the ex-Gaussian model (*r* = −0.34, *p* = 0.01, *Pearson*’s correlation, Fig. 1C).

Dimensional analyses revealed that drift rate (*v*) significantly correlated with inattention scores (*r* = −0.28, *p* = 0.04) but not with hyperactivity/impulsivity scores (*p* > 0.2). Decision boundary (*a*) and non-decision time (*t*) were not significantly correlated with either attentional score (*p*s > 0.05). These results suggest that inattention, rather than hyperactivity/impulsivity, is associated with evidence accumulation speed.

### Latent decision-making processes: categorical analysis

Categorical analyses revealed significant between-group differences in drift rate (*t* = 2.95, *p* = 0.005, two-sample *t*-test, Fig. 1B) and non-decision time (*t* = 2.7, *p* = 0.01, two-sample *t*-test), but no significant difference in decision boundary (*p* = 0.2), indicating that children with ADHD have slower evidence accumulation speed than TD children.

### Dynamic brain states: dimensional analysis

To probe dynamic brain states supporting sustained attention, we focused on circuits associated with the SN, FPN, and DMN, including bilateral anterior insula, middle frontal gyrus (MFG), frontal eye fields (FEFs), inferior parietal lobe, PCC, VMPFC, and pre supplementary motor area (Fig. 1D). These ROIs were determined using an independent study demonstrating strong attentional load effects in each of these regions [38]. Then we applied BSDS (Fig. 1E) on the time series extracted from the ROIs. BSDS uncovered four different latent brain states from the simple choice response task labeled S1, S2, S3, and S4 (Fig. 1F, G). The occupancy rate, which measures how often a latent brain state occurs during the task, and mean lifetime, which measures dwelling time of a brain state before switching to another state, were used to assess temporal properties of each latent brain state.

Dimensional analyses revealed that the occupancy rate of S2 was positively and significantly correlated with inattention (*r* = 0.27, *p* = 0.04), but this result was not significant after multiple comparisons correction. Mean lifetime of S2 was not correlated with inattention, and both occupancy and mean lifetime of S2 were not correlated with hyperactivity/impulsivity (*p*s > 0.05). No other brain state’s occupancy rate and mean lifetimes significantly correlated with either score (*p*s > 0.05).

### Dynamic brain states: categorical analysis

Occupancy rates of S1 were greater in TD children than children with ADHD (ADHD: 15 ± 8%, TD: 21 ± 9%, *p* < 0.05 FDR corrected, two-sample *t*-test), occupancy rates of S2 were greater in children with ADHD than TD children (ADHD: 36 ± 13%, TD: 27 ± 10%, *p* < 0.05 FDR corrected, two-sample t-test), but occupancy rates of S3 or S4 did not differ (*ps* > 0.5). There was no significant between-group difference in mean lifetimes of any brain state (*ps* > 0.05). These results suggest that S1 and S2 are the most clinically relevant latent brain states. Thus, we focus on these two states in the following analyses.

### Dynamic brain states in relation to IIRV

**Fig. 2:**
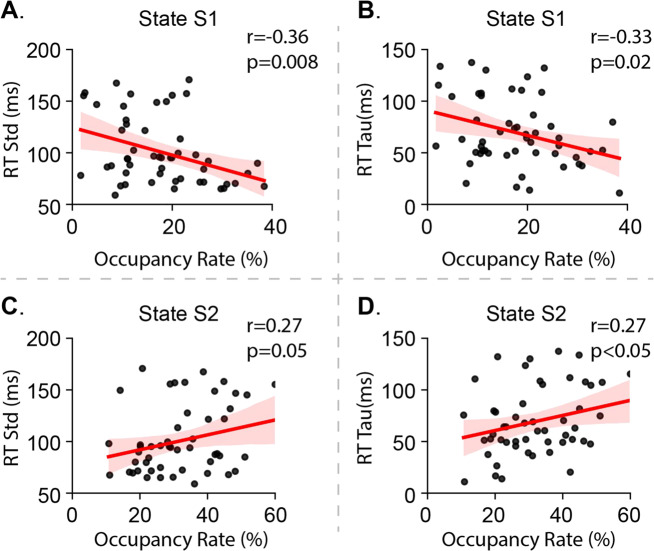
Occupancy rate of latent brain state in relation to IIRV. **A** The occupancy rate (OR) of latent brain state S1 is negatively correlated with RT std. **B** OR of latent brain state S1 is negatively correlated with RT tau. **C** OR of latent brain state S2 is positively correlated with RT std. **D** OR of latent brain state S2 is positively correlated with RT tau.

We tested whether engaging in latent brain states S1 or S2 altered IIRV during task performance. The occupancy rate of S1 negatively correlated with RT std (*r* = −0.35, *p* = 0.008, Fig. 2A) and with RT tau (*r* = −0.33, *p* = 0.02, Fig. 2B). In contrast, the occupancy rate of S2 was marginally positively correlated with RT std (*r* = 0.27, *p* = 0.05, Fig. 2C) and RT tau (*r* = 0.27, *p* = 0.046, Fig. 2D). Mean lifetimes of latent brain states S1 and S2 were not significantly correlated with RT std or RT tau (*p*s > 0.1). Additional multiple linear regression analysis using age, gender, IQ, and head motion as covariates confirmed that the occupancy rate of S1 and S2 contributed unique variance and emerged as the dominant predictors for both RT std and RT tau (Table S2). These findings suggest that engagement of S1 boosts stable behavioral performance, whereas engagement of S2 undermines stable performance during a simple choice response task.

### Dynamic brain states in relation to decision-making processes

**Fig. 3:**
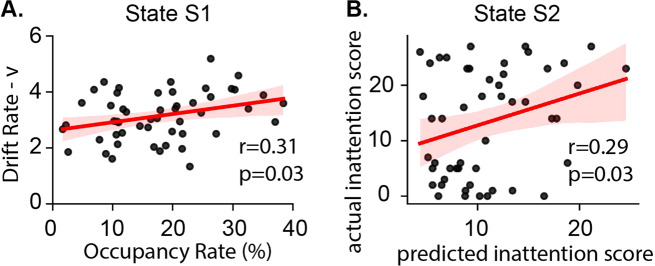
Occupancy rate of latent brain state in relation to deicsion-making and inattention. **A** The Occupancy rate of latent brain state S1 is positively correlated with evidence accumulation speed (*v*). **B** The Occupancy rate and mean lifetime of latent brain state S2 predict inattention scores.

We then examined dynamic brain states in relation to decision-making processes. The occupancy rate of S1 positively correlated with drift rate (*v*) (*r* = 0.31, *p* = 0.03, Fig. 3A) but not with decision boundary (*a*) or non-decision time (*t*) (*p*s > 0.05). Mean lifetime of S1 was not significantly correlated with any latent component in the decision-making model (*p*s > 0.1). S2 was not correlated with any decision-making parameters (*p*s > 0.05). Additional multiple linear regression analysis using age, gender, IQ, and head motion as confounds confirmed that the occupancy rate of S1 was the only significant predictor for drift rate (*v*) (Table S3). Findings suggest that engaging S1 facilitates information accumulation speed and promotes faster and accurate responses.

### Brain states dynamics predict inattention symptoms

Next, we examined whether occupancy rate and mean lifetime of a latent brain state would predict inattention and hyperactivity/impulsivity, core symptoms of ADHD. We trained nonlinear support vector regression (SVR) models based on the occupancy rate and mean lifetime of each latent brain state, separately. Latent brain state S2 accurately predicted inattention scores (*r* = 0.29, *p* = 0.03, Fig. 3B**)**. No other latent brain state predicted inattention scores and no brain state predicted hyperactivity/impulsivity scores (Table S4).

### Distinct functional connectivity patterns underlying latent brain states

**Fig. 4:**
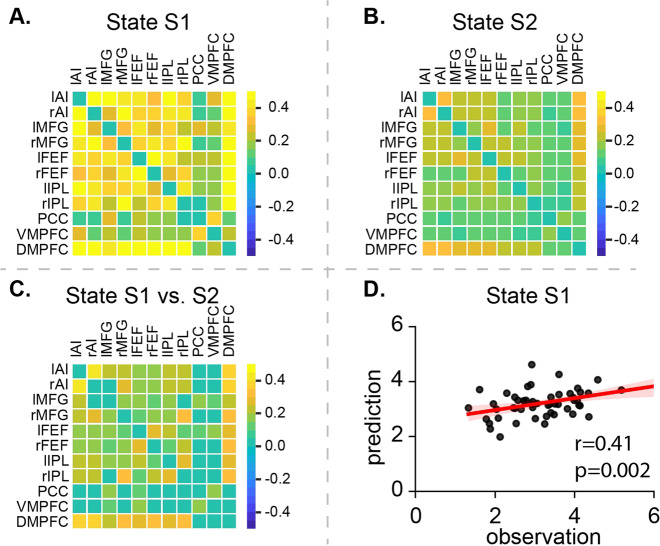
Functional connectivity of latent brain state and its relation to decision-making. **A** Latent brain states S1 and **B** S2 were characterized by their distinct functional connectivity. **C** Link-by-link analysis showed that strong connectivity within and between key nodes in SN and FPN in S1 than S2 (*p* < 0.001, FDR corrected). **D** Multivariate functional connectivity patterns in S1 accurately predict drift rate (*v*) of the DDM during the simple choice response task (*r* = 0.41, *p* = 0.002).

Next, we examined the patterns of functional connectivity between regions in the SN, FPN, and DMN associated with latent brain states (Fig. 4A, B). S1 had a significantly stronger interaction between brain regions in the SN and FPN than S2 (*p* < 0.001, FDR corrected, Fig. 4C).

### SN-FPN functional connectivity predicts evidence accumulation

To further understand the relationship between functional connectivity in latent state S1 and the positive contribution of S1 in behavioral stability and decision-making processes, we fit multivariate patterns of functional connectivity of S1 and IIRV (e.g., tau) and drift rate in the Lasso and Elastic-Net Regularized General Linear Models [53] with leave-one-out cross validation (LOOCV). To reduce dimensionality, only functional connections that significantly differentiated S1 and S2 (Fig. 4C) were included in the prediction model. Functional connectivity in S1 accurately predicted drift rate in children (*r* = 0.41, *p* = 0.002, Fig. 4D) but not IIRV (*p* > 0.05). These effects were specific to the drift rate, as the same model and features did not predict inattention scores (*p* > 0.5). Our findings suggest that multivariate patterns of functional connectivity in SN and FPN are robust predictors of evidence accumulation during decision-making processes associated with task performance.

### SN-DMN functional connectivity predicts inattention

**Fig. 5:**
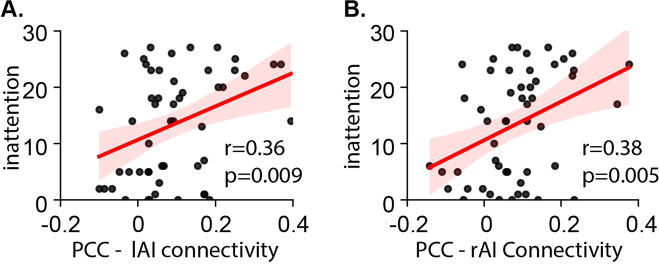
Functional connectivity of PCC-AI in relation to inattention. **A** Inattention scores were associated with functional connectivity between PCC and lAI, and **B** PCC and rAI in latent brain S2 (*p*s < 0.05, FDR corrected).

Based on reports that functional connectivity between PCC and task-activated regions is associated with attention problems [32–34, 54], we examined functional connectivity between PCC and individual nodes of the SN and FPN. We first focused on latent state S2 as its occupancy rate was negatively correlated with inattention, and evaluated individual PCC links with SN and FPN nodes in relation to inattention symptoms. Children’s inattention scores significantly correlated with functional connectivity of PCC-left anterior insula (lAI; *r* = 0.36, *p* = 0.009, *Pearson*’s correlation, Fig. 5A) and PCC-right AI (rAI; *r* = 0.38, *p* = 0.005, *Pearson*’s correlation, Fig. 5B), and were significant after multiple comparisons correction (*p*s < 0.05, FDR corrected). A similar pattern of results was also observed in state S1 (Fig. S1), suggesting that the inability of the SN to disentangle from the DMN is associated with greater severity of inattention symptoms across both non-optimal and optimal latent brain states.

### Brain state dynamics distinguish children with ADHD from TD children

Finally, we conducted categorical analyses to determine whether latent brain states could distinguish children with ADHD from TD children. We used support vector machine (SVM) classification analysis with the occupancy rate and mean lifetime of each latent state. Only latent state S2, a non-optimal brain state, distinguished children with ADHD from TD children (CV ACC = 65%, *p* = 0.03, Table S5). Results demonstrate that latent task-related brain state dynamics distinguish children with ADHD from TD children.

## Discussion

Deficits in attention are common in neurodevelopmental disorders, particularly in ADHD, manifesting as elevated response variability and poor decision-making. However, the dynamic brain mechanisms of these deficits are poorly understood. Using ultrafast task-fMRI, we identified multiple latent brain states during a simple choice response task and related it to IIRV, latent decision-making processes, and inattention using an RDoC approach. We applied a novel unsupervised learning algorithm on fMRI data to uncover latent brain state dynamics during cognitive performance and found that occupancy rates of a task-optimal latent brain state were significantly correlated with IIRV and information accumulation speed estimated from a decision-making model. In addition, dynamic properties of a non-optimal latent brain state predicted inattention scores. Furthermore, functional connectivity patterns associated with SN and FPN predicted drift rate, whereas functional connectivity between SN and DMN nodes, AI and PCC, predicted inattention scores. To identify clinically significant phenomena, we conducted complementary categorical analyses. Children with ADHD had larger IIRV, slower information accumulation speed, and lower occupancy rate of the task-optimal brain state than TD children, and the dynamic properties of the non-optimal brain state distinguished children with ADHD from TD children. Together, findings demonstrate that dissociable latent brain state dynamics distinguish behavioral variability, slow decision-making processes, and inattention symptoms, thus advancing our understanding of the mechanisms associated with attention deficits in children.

### High IIRV: relation to inattention and childhood ADHD

High IIRV is one intermediate phenotype of childhood ADHD, although the nature of this variability is not known. Compared with TD children, children with ADHD display increased IIRV during cognitive performance [46, 49]. Although the standard deviation is typically used to measure IIRV, the ex-Gaussian distribution is recommended to identify the exponential component in the RT distribution, tau, which has been linked to attentional lapse [36, 55]. Tau measures the positive tail of a skewed normal distribution, reflecting an increase in frequency and magnitude of extremely slow responses that substantially effect mean and variance estimation of the observed RT. Children with ADHD have larger tau values than TD children, whereas mu and sigma do not consistently differentiate the two groups [5, 56, 57]. Inattention was marginally associated with IIRV using a dimensional approach. Follow-up categorical analyses demonstrated that children with ADHD have larger standard deviations of RT than TD children and that the source of this difference is tau. Our study demonstrates that IIRV is correlated with inattention rather than hyperactivity/impulsivity symptoms, which is aligned with profiles of attentional deficits reported in children with ADHD [55, 59, 60]. Crucially, a simple choice response task, which does not require overt modulation of attention or cognitive control in a trial-specific manner, reduces the impact of trial-by-trial anticipation and feedback-induced response strategy adjustment on intra-individual variability, providing a more specific estimation of IIRV associated with sustained attention.

### Slow decision-making processes are associated with inattention and childhood ADHD

Although substantial behavioral variability is commonly manifested in individuals with attention deficits, overt IIRV measures cannot uncover latent cognitive sources of behavioral instability. We used a HDDM to determine whether inattention and/or hyperactivity/impulsivity are distinguished by decision-making processes associated with behavioral instability. Drift-diffusion models quantify decision-making processes by estimating three latent components underlying the time to respond on each trial: (1) drift rate, which indexes how fast evidence is accumulated for a decision, (2) decision threshold, which indexes the distance to a decision boundary, and (3) a non-decision time, which indexes encoding time prior to decision-making [52]. Consistent with hypotheses, inattention was associated with the lower drift rate, reflecting slower, noisier evidence accumulation. Complementary categorical analyses determined that children with ADHD are much slower in accumulating information for decision-making (lower drift rate) than TD children. Our findings are consistent with previous studies showing ADHD undermines decision-making processes [61, 62] and extends prior work by demonstrating that inattention rather than hyperactivity/impulsivity, at least within the context of a simple choice RT task, drives these effects. Theoretical frameworks of ex-Gaussian and diffusion drift models of RT have suggested correspondence between model parameters [58]. We found that tau of the ex-Gaussian model, reflecting extremely slow responses, was negatively correlated with drift rate but not with decision threshold, suggesting that poor control of sustained attention, rather than impulsive responding, underlies behavioral instability.

### Dynamic latent brain states underlying response variability

Elevated response variability is associated with deficits in sustained attention in children with ADHD [5, 56, 57, 63] and variability in neural signals [7, 64]. Activity and connectivity in DMN and cognitive control regions are related to performance fluctuation during cognitive performance and deficits in sustained attention [65, 66]. The present study moved beyond regional activity and investigated latent brain states characterized by multivariate patterns of brain activity and functional connectivity using a novel BSDS unsupervised learning algorithm. Our findings uncover an optimal latent brain state (S1), the occurrence of which predicts response variability. Furthermore, we showed that this relation can be replicated using overt and latent behavioral measures associated with attention deficits, but its dysfunctional neural mechanisms remain unknown. Brain areas in the SN, FPN, and DMN implicated in decision-making and executive function are known to show altered activation and connectivity patterns in children with ADHD [6, 31, 32, 34, 67, 68]. However, regional activity and connectivity are insufficient to represent the complexity and multidimensional nature of decision-making processes as individuals’ trait-like characteristics (e.g., how impulsive one is) are also affected by state-like factors (e.g., fatigue, attentional lapse) across time. To address this challenge, we identified dynamic brain states characterized by multivariate patterns of activation and functional connectivity in SN, FPN, and DMN regions in an optimal latent space and investigated temporal properties of latent brain states in relation to model parameters that represent decision-making components. As predicted, difficulty engaging in a task-optimal latent brain state jeopardizes information accumulation speed and undermines the decision-making process, recapitulating previous findings in an n-back working memory study of neurotypical adults [38].

### Dynamic latent brain states in relation to inattention and childhood ADHD

Attentional deficits are a defining symptom of neurodevelopmental disorders, particularly ADHD. An influential neurobiological model hypothesizes that a task-non-specific brain state can intrude on a task-specific brain state during cognitive performance and lead to fluctuations in attention [7]. Although some studies have shown that abnormal activity and connectivity in the DMN is associated with attentional deficits [32], robust evidence linking a task-non-specific brain state with attention problems is lacking. Several recent studies have tested this hypothesis using resting-state fMRI data [6, 69], but little work has been done on task-fMRI data. We used task-based fMRI and tested the contribution of a non-optimal latent brain state to behavioral performance, sustained attention, and hyperactivity/impulsivity. Our analyses determined that the latent brain state S2 is highly relevant to poor behavioral performance and problematic sustained attention. First, increased occupancy rate of S2 was associated with increased response variability, suggesting that engagement of S2 will lead to behavioral instability. Second, occupancy rates and mean lifetimes of S2 predicted inattention symptoms. Third, categorical analyses determined that occupancy rates and mean lifetimes of S2 could successfully distinguish children with ADHD from TD children. Notably, no other latent brain state could successfully predict inattention symptoms or classify children with ADHD and TD children, suggesting the unique clinical relevance of S2 in childhood ADHD. Furthermore, dynamic profiles of S2 significantly predicted inattention but not hyperactivity/impulsivity scores, suggesting the specific relevance of brain state S2 to inattention.

### SN, FPN, and DMN connectivity in relation to decision-making and inattention

Each latent brain state was characterized by distinct patterns of interregional connectivity. Brain state S1, which has a positive effect on behavioral stability, was associated with increased connectivity between regions in the SN and FPN, compared to brain state S2, which has a negative effect on behavioral stability and sustained attention. This finding converges on a previous observation that reduced task-modulated connectivity between SN and FPN is associated with attention problems and childhood ADHD [31]. Crucially, our analyses have not only uncovered unique connectivity patterns that differentiate latent brain states S1 and S2, but also identified functional connections that are associated with decision-making processes and attention problems. Using a prediction model with cross-validation, we demonstrated that multivariate patterns of functional connectivity in SN and FPN accurately predicted drift rates of decision-making processes. Increased interactions in SN and FPN are associated with high load cognitive processes in adults [38, 43, 70, 71] and developmental groups [31]. SN plays a crucial role in switching interactions with large-scale brain networks and facilitating access to attention resources [72]. In particular, AI, the key node of the SN, is implicated in signal detection during attentionally demanding tasks [10, 18, 43]. In contrast, functional connectivity between PCC and AI is correlated with inattention scores in children. These data are consistent with previous studies showing that intrinsic connectivity between DMN regions and SN regions is correlated with attention problems [32, 33, 73]. Interactions between regions in SN and DMN have been associated with attentional modulation [74, 75]. Our findings reveal dissociable brain–behavior relationships such that functional connectivity of SN and FPN is related to latent decision-making processes, whereas functional connectivity between SN and DMN underlies inattention.

## Clinical implications

Across development, symptoms of inattention are more likely to remain stable, whereas symptoms of hyperactivity/impulsivity are more likely to decline [76]. However, the contextual and etiological determinants of this developmental divergence and possible transition to psychopathology is markedly underspecified. Our findings demonstrate broad effects of inattention in relation to behavioral instability, poor decision-making, a suboptimal brain state, and functional connectivity between key regions in the SN and DMN. The tendency to engage in a non-optimal brain state that undermines behavioral stability and sustained attention may be a discriminating feature of attention deficits and has broad appeal for neurodevelopmental disorders in general. Moreover, taking a dynamic approach to understanding symptom presentation and fluctuation may resolve ADHD heterogeneity. Our findings may provide promising targets for understanding atypical developmental trajectories and identifying etiological contributions to ADHD.

## Conclusion

Our study provides new insights into latent brain state dynamics that give rise to behavioral instability and inattention in children, linking observable disruptions in clinical phenomena associated with ADHD. We applied a novel switching dynamical system approach for investigating latent brain mechanisms underlying behavioral instability, poor decision-making, and deficits in attention. Engagement of a task-optimal latent brain state facilitated stable behavioral performance and information accumulation speed, whereas dynamic profiles of a non-optimal latent brain state predicted inattention symptoms in a dimensional analysis and differentiated children with ADHD from TD children in a categorical analysis. Functional connectivity between SN, FPN, and DMN regions, three core cognitive control networks implicated in psychopathology [77], differentiated latent brain states and was associated with distinct latent and observed features of behavioral problems. These findings advance our understanding of the latent dynamic processes in relation to dimensions of behavioral instability and inattention that in excess, play a significant role in childhood ADHD.

## Methods

### **P**articipants

One hundred and seven children (9–12 years old) were recruited from the local community. Informed consent was obtained from legal guardians of the children and the study was approved by the Institutional Review Board of Stanford University. Ninety children completed a choice response task in the scanner. Thirty-eight children were excluded in the analysis because of missing data, image artifacts, excessive head motion, and behavioral outliers (see details below). The final dataset included 29 children with ADHD (11 F/18 M) and 23 TD children (11 F/12 M).

### Clinical and neuropsychological assessments

Children and their guardians completed a clinical and neuropsychological assessment session. ADHD diagnosis was informed by children’s guardians and further confirmed using the Attention Deficit/Hyperactivity Disorder Rating Scale (ADHD-RS). Inclusion criterion were the following: no history of claustrophobia, head injury, serious neurological or medical illness, autism, psychosis, mania/bipolar, major depression, learning disability, substance abuse, sensory impairment such as vision or hearing loss, birth weight less than 2000 g, and/or gestational ages of less than 34 weeks. All children were right-handed with an IQ greater than 80. For all children, inattention and hyperactivity/impulsivity symptoms were assessed using the ADHD-RS. Although clinical presentations of ADHD were determined, the small sample size in each presentation group prevented further analysis of the specificity of each subtype. All participants were free of medication during testing and a washout period of at least 5 half-lives was applied.

### Choice response task

In the choice response task, a left- or right-pointing arrow was presented for 500 ms on each trial. Participants were told to press a left or right button based on the direction of the arrow within a 1.5 s response window after the onset of the stimulus. One task run included 96 trials with jittered inter-trial-intervals between 1 and 4 s. Each participant completed one run of the choice response task during scanning. Behavioral outliers were defined by 2.5 standard deviation of key measures, including accuracy and RT.

### Behavioral analysis: ex-Gaussian model

An ex-Gaussian toolbox implemented in Matlab [78] was used to fit the RT data with maximum likelihood estimation and estimate parameters (mu, sigma, and tau) for each participant. The ex-Gaussian model includes three parameters: mu, sigma and tau. Mu and sigma are the mean and std in the Gaussian component, respectively. Tau represents the exponential component, which captures the positive tail of the skewed normal distribution.

### Behavioral analysis: HDDM model

HDDM [51] estimated parameters *a*, *v*, and *t*. See Supplementary Method for details.

### MRI acquisition

MRI data were acquired on a 3T GE Signa scanner using a 32-channel head coil at the Richard M Lucas Center for Imaging at Stanford University. Functional images of 42 axial slices were acquired using the multiband gradient-echo planar imaging with the following parameters: TR = 490 ms; TE = 30 ms; flip angle = 45°, FOV = 22.2 cm, matrix = 74 × 74 and in-plane resolution = 3 mm. See Supplementary Method for details.

### fMRI preprocessing

Functional MRI data were preprocessed using SPM12 (http://www.fil.ion.ucl.ac.uk/spm/software/spm12). The preprocessing pipeline included realignment, slice-timing correction, co-registration, normalization to MNI space, and smoothing using a 6 mm full-width half-maximum Gaussian kernel to decrease spatial noise. Participants whose maximum displacement exceeded 5 mm in either run were excluded from the analysis and all subjects’ mean scan-to-scan movement were less than 0.5 mm [10].

### Region of interest (ROI) and time series

Eleven ROIs were determined from a previous study of attention and cognitive control [38], including bilateral anterior insula (AI), bilateral MFG, bilateral FEF, bilateral intraparietal sulcus, dorsomedial prefrontal cortex, VMPFC, and PCC. Each ROI was 6-mm radius sphere centered at the peak voxel.

Time series of the 1st eigenvalue was extracted from each ROI per subject. A multiple linear regression approach with six realignment parameters (three translations and three rotations) was applied to each time series to reduce head-motion-related artifacts; the resulting time series was further high-pass filtered (*f* > 0.008 Hz).

### BSDS model

We used a BSDS model [38] to uncover latent brain states during cognitive performance of the simple choice response task. A brief explanation of the BSDS model is in Supplementary Method. Detailed theoretical derivations are provided in our previous study [38].

Key measures extracted from BSDS include occupancy rate, mean lifetime of latent brain state and covariance of states.

### Brain state dynamics in relation to behavioral performance

To understand the relationship between the same brain state and behavioral performance in different tasks, we examined whether occupancy rates and mean lifetime of each brain is related to behavioral performance.

### Brain state dynamics predict dimensions of attention and hyperactivity/impulsivity

To examine whether state dynamics could account for individual differences in core symptoms of ADHD, we conducted multivariate regression analysis using nonlinear SVR. The occupancy rate and mean lifetimes on each latent brain state were used as features to predict inattention or hyperactivity/impulsivity scores, separately. The model was evaluated using the aforementioned LOOCV. *Pearson*’s correlations were used to evaluate correspondence between predicted values and observed values.

### Functional connectivity of time-varying latent brain states

To determine which dynamic functional connections are important for distinguishing different brain states, we conducted paired *t*-tests on the covariance matrix between latent brain states derived from BSDS analysis. Multiple comparisons were corrected using false discovery rate (*p* < 0.001).

### Functional connectivity predicts IIRV, drift rate, and inattention

To test whether functional connectivity patterns in latent brain states predicted IIRV, drift rate, or inattention scores, we conducted a prediction analysis using the Lasso and Elastic-Net Regularized General Linear Model [53]. See Supplementary Method for details of the prediction model.

### Brain state dynamics differentiate TD and ADHD children

To examine whether brain state dynamics could successfully differentiate TD children and children with ADHD, we conducted multivariate classification analysis using linear SVM. The occupancy rate and mean lifetimes of each latent brain state were used as features to predict group identity of each child (TD or ADHD), separately. See Supplementary Method for more details.

## Supplementary information

Supplementary Material

## Data Availability

All codes are available upon request.

## References

[1] Polanczyk G, de Lima MS, Horta BL, Biederman J, Rohde LA (2007). The worldwide prevalence of ADHD: a systematic review and metaregression analysis. Am J Psychiatry.

[2] Kofler MJ, Rapport MD, Sarver DE, Raiker JS, Orban SA, Friedman LM (2013). Reaction time variability in ADHD: a meta-analytic review of 319 studies. Clin Psychol Rev.

[3] Lijffijt M, Kenemans JL, Verbaten MN, van Engeland H (2005). A meta-analytic review of stopping performance in attention-deficit/hyperactivity disorder: deficient inhibitory motor control?. J Abnorm Psychol.

[4] Castellanos FX, Sonuga-Barke EJ, Scheres A, Di Martino A, Hyde C, Walters JR (2005). Varieties of attention-deficit/hyperactivity disorder-related intra-individual variability. Biol Psychiatry.

[5] Vaurio RG, Simmonds DJ, Mostofsky SH (2009). Increased intra-individual reaction time variability in attention-deficit/hyperactivity disorder across response inhibition tasks with different cognitive demands. Neuropsychologia.

[6] Cai W, Chen T, Szegletes L, Supekar K, Menon V (2018). Aberrant time-varying cross-network interactions in children with attention-deficit/hyperactivity disorder and the relation to attention deficits. Biol Psychiatry Cogn Neurosci Neuroimaging.

[7] Sonuga-Barke EJ, Castellanos FX (2007). Spontaneous attentional fluctuations in impaired states and pathological conditions: a neurobiological hypothesis. Neurosci Biobehav Rev.

[8] Cuthbert BN, Insel TR (2010). Toward new approaches to psychotic disorders: the NIMH Research Domain Criteria project. Schizophr Bull.

[9] Musser ED, Raiker JS (2019). Attention-deficit/hyperactivity disorder: an integrated developmental psychopathology and Research Domain Criteria (RDoC) approach. Compr Psychiatry.

[10] Cai W, Duberg K, Padmanabhan A, Rehert R, Bradley T, Carrion V (2019). Hyperdirect insula-basal-ganglia pathway and adult-like maturity of global brain responses predict inhibitory control in children. Nat Commun.

[11] Crittenden BM, Mitchell DJ, Duncan J (2017). Task encoding across the multiple demand cortex is consistent with a frontoparietal and cingulo-opercular dual networks distinction (vol 36, pg 6147, 2017). J Neurosci.

[12] Cole MW, Bassett DS, Power JD, Braver TS, Petersen SE (2014). Intrinsic and task-evoked network architectures of the human brain. Neuron.

[13] Cole MW, Ito T, Bassett DS, Schultz DH (2016). Activity flow over resting-state networks shapes cognitive task activations. Nat Neurosci.

[14] Dove A, Pollmann S, Schubert T, Wiggins CJ, von Cramon DY (2000). Prefrontal cortex activation in task switching: an event-related fMRI study. Brain Res Cogn Brain Res.

[15] Leung HC, Skudlarski P, Gatenby JC, Peterson BS, Gore JC (2000). An event-related functional MRI study of the stroop color word interference task. Cereb cortex.

[16] Leung HC, Cai W (2007). Common and differential ventrolateral prefrontal activity during inhibition of hand and eye movements. J Neurosci.

[17] McNab F, Leroux G, Strand F, Thorell L, Bergman S, Klingberg T (2008). Common and unique components of inhibition and working memory: an fMRI, within-subjects investigation. Neuropsychologia.

[18] Cai W, Ryali S, Chen T, Li CS, Menon V (2014). Dissociable roles of right inferior frontal cortex and anterior insula in inhibitory control: evidence from intrinsic and task-related functional parcellation, connectivity, and response profile analyses across multiple datasets. J Neurosci.

[19] Levy BJ, Wagner AD (2011). Cognitive control and right ventrolateral prefrontal cortex: reflexive reorienting, motor inhibition, and action updating. Ann N Y Acad Sci.

[20] Owen AM, McMillan KM, Laird AR, Bullmore E (2005). N-back working memory paradigm: a meta-analysis of normative functional neuroimaging studies. Hum Brain Mapp.

[21] Swick D, Ashley V, Turken U (2011). Are the neural correlates of stopping and not going identical? Quantitative meta-analysis of two response inhibition tasks. NeuroImage.

[22] Wager TD, Sylvester CY, Lacey SC, Nee DE, Franklin M, Jonides J (2005). Common and unique components of response inhibition revealed by fMRI. NeuroImage.

[23] Anticevic A, Repovs G, Shulman GL, Barch DM (2010). When less is more: TPJ and default network deactivation during encoding predicts working memory performance. NeuroImage.

[24] Arsenault JT, Caspari N, Vandenberghe R, Vanduffel W (2018). Attention shifts recruit the monkey default mode network. J Neurosci.

[25] Crittenden BM, Mitchell DJ, Duncan J (2015). Recruitment of the default mode network during a demanding act of executive control. Elife.

[26] Hampson M, Driesen NR, Skudlarski P, Gore JC, Constable RT (2006). Brain connectivity related to working memory performance. J Neurosci.

[27] Cortese S, Kelly C, Chabernaud C, Proal E, Di Martino A, Milham MP (2012). Toward systems neuroscience of ADHD: a meta-analysis of 55 fMRI studies. Am J Psychiatry.

[28] Dickstein SG, Bannon K, Castellanos FX, Milham MP (2006). The neural correlates of attention deficit hyperactivity disorder: an ALE meta-analysis. J Child Psychol Psychiatry.

[29] Hart H, Radua J, Mataix-Cols D, Rubia K (2012). Meta-analysis of fMRI studies of timing in attention-deficit hyperactivity disorder (ADHD). Neurosci Biobehav Rev.

[30] Norman LJ, Carlisi C, Lukito S, Hart H, Mataix-Cols D, Radua J (2016). Structural and functional brain abnormalities in attention-deficit/hyperactivity disorder and obsessive-compulsive disorder: a comparative meta-analysis. JAMA Psychiatry.

[31] Cai W, Griffiths K, Korgaonkar MS, Williams LM, Menon V. Inhibition-related modulation of salience and frontoparietal networks predicts cognitive control ability and inattention symptoms in children with ADHD. Mol Psychiatry. 2019;1–10. 10.1038/s41380-019-0564-4 PMC7188596 31664176

[32] Castellanos FX, Margulies DS, Kelly C, Uddin LQ, Ghaffari M, Kirsch A (2008). Cingulate-precuneus interactions: a new locus of dysfunction in adult attention-deficit/hyperactivity disorder. Biol Psychiatry.

[33] Elton A, Alcauter S, Gao W (2014). Network connectivity abnormality profile supports a categorical-dimensional hybrid model of ADHD. Hum Brain Mapp.

[34] Fair DA, Posner J, Nagel BJ, Bathula D, Dias TG, Mills KL (2010). Atypical default network connectivity in youth with attention-deficit/hyperactivity disorder. Biol Psychiatry.

[35] Epstein JN, Hwang ME, Antonini T, Langberg JM, Altaye M, Arnold LE (2010). Examining predictors of reaction times in children with ADHD and normal controls. J Int Neuropsychol Soc.

[36] Tamm L, Narad ME, Antonini TN, O’Brien KM, Hawk LW, Epstein JN (2012). Reaction time variability in ADHD: a review. Neurotherapeutics.

[37] Smith PL, Ratcliff R (2009). An integrated theory of attention and decision making in visual signal detection. Psychol Rev.

[38] Taghia J, Cai WD, Ryali S, Kochalka J, Nicholas J, Chen TW (2018). Uncovering hidden brain state dynamics that regulate performance and decision-making during cognition. Nat Commun.

[39] Braun U, Schafer A, Walter H, Erk S, Romanczuk-Seiferth N, Haddad L (2015). Dynamic reconfiguration of frontal brain networks during executive cognition in humans. Proc Natl Acad Sci USA.

[40] Kitzbichler MG, Henson RN, Smith ML, Nathan PJ, Bullmore ET (2011). Cognitive effort drives workspace configuration of human brain functional networks. J Neurosci.

[41] Shine JM, Bissett PG, Bell PT, Koyejo O, Balsters JH, Gorgolewski KJ (2016). The dynamics of functional brain networks: integrated network states during cognitive task performance. Neuron.

[42] Hutchison RM, Womelsdorf T, Allen EA, Bandettini PA, Calhoun VD, Corbetta M (2013). Dynamic functional connectivity: promise, issues, and interpretations. NeuroImage.

[43] Cai W, Chen T, Ide JS, Li CR, Menon V (2017). Dissociable fronto-operculum-insula control signals for anticipation and detection of inhibitory sensory cue. Cereb Cortex.

[44] Shenoy P, Yu AJ (2011). Rational decision-making in inhibitory control. Front Hum Neurosci.

[45] Ceccarini F, Castiello U (2018). The grasping side of post-error slowing. Cognition.

[46] Klein C, Wendling K, Huettner P, Ruder H, Peper M (2006). Intra-subject variability in attention-deficit hyperactivity disorder. Biol Psychiatry.

[47] Ratcliff R, Murdock BB (1976). Retrieval processes in recognition memory. Psychol Rev.

[48] Heathcote A, Popiel SJ, Mewhort DJK (1991). Analysis of response-time distributions - an example using the stroop task. Psychol Bull.

[49] Kuntsi J, Klein C (2012). Intraindividual variability in ADHD and its implications for research of causal links. Curr Top. Behav Neurosci.

[50] Willcutt EG, Doyle AE, Nigg JT, Faraone SV, Pennington BF (2005). Validity of the executive function theory of attention-deficit/hyperactivity disorder: a meta-analytic review. Biol Psychiatry.

[51] Wiecki TV, Sofer I, Frank MJ (2013). HDDM: hierarchical bayesian estimation of the drift-diffusion model in python. Front Neuroinform.

[52] Ratcliff R, McKoon G (2008). The diffusion decision model: theory and data for two-choice decision tasks. Neural Comput.

[53] Friedman J, Hastie T, Tibshirani R (2010). Regularization paths for generalized linear models via coordinate descent. J Stat Softw.

[54] Hoekzema E, Carmona S, Ramos-Quiroga JA, Fernandez VR, Bosch R, Soliva JC (2014). An independent components and functional connectivity analysis of resting state FMRI data points to neural network dysregulation in adult ADHD. Hum Brain Mapp.

[55] Castellanos FX, Sonuga-Barke EJS, Milham MP, Tannock R (2006). Characterizing cognition in ADHD: beyond executive dysfunction. Trends Cogn Sci.

[56] Hervey AS, Epstein JN, Curry JF, Tonev S, Eugene Arnold L, Keith (2006). Reaction time distribution analysis of neuropsychological performance in an ADHD sample. Child Neuropsychol.

[57] Leth-Steensen C, Elbaz ZK, Douglas VI (2000). Mean response times, variability, and skew in the responding of ADHD children: a response time distributional approach. Acta Psychol.

[58] Schmiedek F, Oberauer K, Wilhelm O, Suss HM, Wittmann WW (2007). Individual differences in components of reaction time distributions and their relations to working memory and intelligence. J Exp Psychol Gen.

[59] Hinshaw SP (2018). Attention deficit hyperactivity disorder (ADHD): controversy, developmental mechanisms, and multiple levels of analysis. Annu Rev Clin Psychol.

[60] Nigg JT, Stavro G, Ettenhofer M, Hambrick DZ, Miller T, Henderson JM (2005). Executive functions and ADHD in adults: evidence for selective effects on ADHD symptom domains. J Abnorm Psychol.

[61] Karalunas SL, Huang-Pollock CL, Nigg JT (2012). Decomposing attention-deficit/hyperactivity disorder (ADHD)-related effects in response speed and variability. Neuropsychology.

[62] Metin B, Roeyers H, Wiersema JR, van der Meere JJ, Thompson M, Sonuga-Barke EJS (2013). ADHD Performance reflects inefficient but not impulsive information processing: a diffusion model analysis. Neuropsychology.

[63] Castellanos FX, Tannock R (2002). Neuroscience of attention-deficit/hyperactivity disorder: the search for endophenotypes. Nat Rev Neurosci.

[64] Gonen-Yaacovi G, Arazi A, Shahar N, Karmon A, Haar S, Meiran N (2016). Increased ongoing neural variability in ADHD. Cortex.

[65] Bonnelle V, Leech R, Kinnunen KM, Ham TE, Beckmann CF, De Boissezon X (2011). Default mode network connectivity predicts sustained attention deficits after traumatic brain injury. J Neurosci.

[66] Esterman M, Noonan SK, Rosenberg M, Degutis J (2013). In the zone or zoning out? Tracking behavioral and neural fluctuations during sustained attention. Cereb Cortex.

[67] Rubia K, Halari R, Smith AB, Mohammad M, Scott S, Brammer MJ (2009). Shared and disorder-specific prefrontal abnormalities in boys with pure attention-deficit/hyperactivity disorder compared to boys with pure CD during interference inhibition and attention allocation. J Child Psychol Psychiatry.

[68] Tomasi D, Volkow ND (2012). Abnormal functional connectivity in children with attention-deficit/hyperactivity disorder. Biol Psychiatry.

[69] Kaboodvand N, Iravani B, Fransson P (2020). Dynamic synergetic configurations of resting-state networks in ADHD. NeuroImage.

[70] Cai WD, Chen TW, Ryali S, Kochalka J, Li CSR, Menon V (2016). Causal interactions within a frontal-cingulate-parietal network during cognitive control: convergent evidence from a multisite-multitask investigation. Cereb Cortex.

[71] Chen T, Cai W, Ryali S, Supekar K, Menon V (2016). Distinct global brain dynamics and spatiotemporal organization of the salience network. PLoS Biol.

[72] Menon V, Uddin LQ (2010). Saliency, switching, attention and control: a network model of insula function. Brain Struct Funct.

[73] Sun L, Cao QJ, Long XY, Sui MQ, Cao XH, Zhu CZ (2012). Abnormal functional connectivity between the anterior cingulate and the default mode network in drug-naive boys with attention deficit hyperactivity disorder. Psychiat Res.

[74] Sridharan D, Levitin DJ, Menon V (2008). A critical role for the right fronto-insular cortex in switching between central-executive and default-mode networks. Proc Natl Acad Sci USA.

[75] Wen XT, Liu YJ, Yao L, Ding MZ (2013). Top-down regulation of default mode activity in spatial visual attention. J Neurosci.

[76] Biederman J, Mick E, Faraone SV (2000). Age-dependent decline of symptoms of attention deficit hyperactivity disorder: Impact of remission definition and symptom type. Am J Psychiatry.

[77] Menon V (2011). Large-scale brain networks and psychopathology: a unifying triple network model. Trends Cogn Sci.

[78] Zandbelt BB. exgauss: A MATLAB toolbox for fitting the ex‐Gaussian distribution to response time data. figshare. 2014.

