# Supplementary material for: Latent brain state dynamics distinguish behavioral variability, impaired decision-making, and inattention
Source: Mol Psychiatry. 2021 Feb 15;26(9):4944–57. doi: 10.1038/s41380-021-01022-3 (PMC8589642; doi:10.1038/s41380-021-01022-3)
Supplement: Supplementary file 1 — Supplementary Material [file 41380_2021_1022_MOESM1_ESM.pdf]

Supplementary Information:

Latent brain state dynamics distinguish behavioral variability, impaired decision-making, and inattention

Weidong Cai, Stacie L. Warren, Katherine Duberg, Bruce Pennington, Stephen Hinshaw, Vinod Menon

Supplementary Methods

Behavioral analysis: HDDM model

The DDM has been extensively used to estimate two-choice decision making processes [1]. In this framework, decisions are modeled as a combination of three parameters: threshold (a) describing the distance between two decision boundaries, drift rate (v) describing the rate at which evidence is accumulated for a given decision, and non-decision time (t) which is representative of those aspects of response time not included in decision making (e.g., stimulus encoding, movement execution). Models will sometimes include a decision bias parameter (z) if there is a reason *a priori* to believe that such bias exists in the task data. As there was no such reason for the simple choice response task, we chose not to model decision bias in this study. Here, we estimated the parameters a, v, and t using the hierarchical DDM (HDDM) [2]. In HDDM, Bayesian inference through Markov chain Monte-Carlo (MCMC) sampling is used to approximate posterior distributions for each parameter at both the individual and group levels. We initialized HDDM to draw 10000 posterior samples for each data with the first 1000 samples discarded as burn-in.

MRI acquisition

MRI data were acquired on a 3T GE Signa scanner using a 32-channel head coil at the Richard M Lucas Center for Imaging at Stanford University. Each participant was instructed to stay as still as possible during the scanning, and inflatable pillows were placed around the child's head in order to further minimize head movement. Functional images of 42 axial slices were acquired using the multiband gradient-echo planar imaging with the following parameters: TR=490ms;

TE=30ms; flip angle=45°, FOV=22.2cm, matrix=74x74 and in-plane resolution=3 mm. A high-order shimming method was used prior to data acquisition to reduce blurring and signal loss arising from field inhomogeneity. High-resolution T1-weighted images were acquired using a spoiled-gradient-recalled inversion recovery three-dimensional (3D) MRI sequence with the following parameters: TR=8.4ms, TE=1.8ms, flip angle=15°, FOV = 22cm, matrix=256x192.

BSDS generative model

Here we briefly describe the BSDS model [3]. Let $\boldsymbol{y}_t^s$ denote a D-dimensional vector of observed fMRI measurements in time t and for subject s. Further, let $\boldsymbol{z}_t^s$ denote a 1-of-K discrete vector of latent state variables of a hidden Markov model (HMM) with elements $z_{kt}^s, \forall k = 1, \ldots, K$. Two consecutive time instances are dependent via a first-order Markov chain through an HMM. Specifically, probability distribution of $\boldsymbol{z}_t^s$ depends on the state of the previous latent variable $\boldsymbol{z}_{t-1}^s$ through a conditional distribution $p(\boldsymbol{z}_t^s \mid \boldsymbol{z}_{t-1}^s, \boldsymbol{A}) = \prod_{k=1}^K \prod_{j=1}^K A_{jk}^{z_{t-1,j}^s \, z_{tk}^s}$ for all $t > 1$ represented by the transition probabilities $\boldsymbol{A}$, where $A_{jk} \equiv p\big(z_{tk}^s = 1 \mid z_{t-1,j}^s = 1\big)$, and a marginal distribution $p(\boldsymbol{z}_1^s \mid \boldsymbol{\pi}) = \prod_{k=1}^K \pi_k^{z_{1k}^s}$ represented by a vector of initial probabilities $\boldsymbol{\pi}$ where $\pi_k \equiv p(z_{1k}^s = 1)$ [4]. Next, we assume that at a given mode of the system given by the latent state $z_{kt}^s = 1$, observed vector $\boldsymbol{y}_t^s$ is generated via a state space model in form of:

$$\boldsymbol{y}_t^s = \boldsymbol{U}_k \boldsymbol{x}_{kt}^s + \boldsymbol{\mu}_k + e_{kt}, \qquad \forall t \mid z_{kt}^s = 1,$$

$$\boldsymbol{x}_{kt}^s = \overline{\boldsymbol{X}}_{kt}^s \vec{\boldsymbol{V}}_k + \boldsymbol{\epsilon}_{kt}, \qquad \forall t \mid z_{kt}^s = 1. \qquad (1)$$

The first line of the generative model in Eq. (1) can be viewed as a probabilistic factor analysis model [5, 6] where $\boldsymbol{U}_k$ is a $D \times P$ dimensional linear transformation matrix that transforms data to a subspace of lower dimensionality, $P < D$, described using a P-dimensional vector of latent space variables $\boldsymbol{x}_{kt}^s$ mediated by an overall bias $\boldsymbol{\mu}_k$ and a measurement noise $\boldsymbol{e}_{kt} \sim \mathcal{N}(\boldsymbol{0}, \boldsymbol{\Psi}_{\boldsymbol{k}})$. The second line of the generative model can be viewed as an autoregressive (AR) process of

order R defined on the latent space variables of the factor analysis model [7]. $\vec{\boldsymbol{V}}_k$ is a vector of AR coefficients. $\overline{\boldsymbol{X}}_{kt}^{s} = \mathrm{diag}(\overline{\boldsymbol{x}}_{kt}^{s})$ is a block diagonal isotropic matrix with elements of $\overline{\boldsymbol{x}}_{kt}^{s} = ({\boldsymbol{x}_{k,t-1}^{s}}^{\mathrm{T}}, {\boldsymbol{x}_{k,t-2}^{s}}^{\mathrm{T}}, \dots, {\boldsymbol{x}_{k,t-R}^{s}}^{\mathrm{T}})$ represented using latent space variables from the previous R time frames where T indicates the transpose operator. $\boldsymbol{\epsilon}_{kt} \sim \mathcal{N}(\boldsymbol{m}_k, \boldsymbol{\Sigma}_k)$ models the remaining error term in the latent space. An AR process of a first order, $R = 1$, is defined on the representations of the observations in the latent subspace, $\boldsymbol{x}_{kt}^{s}$. Detailed theoretical derivations are provided in the previous study [3].

Functional connectivity predicts IIRV, drift rate, and inattention

To examine whether functional connectivity patterns in latent brain states can predict IIRV, drift rate, or inattention scores, we conducted a prediction analysis using the Lasso and Elastic-Net Regularized General Linear Model [8]. The independent variables were functional connectivity values in latent brain state S1 or S2. To reduce dimensionality, only functional connections that were significantly differentiated between S1 and S2 were tested (Figure **5C**). The dependent variables were RT standard deviation of Gaussian model, RT tau of ex-Gaussian model, or inattention scores. Model performance was assessed using LOOCV. *Pearson*'s correlations were used to evaluate correspondence between predicted values and observed values. To further examine functional connectivity between PCC and other SN and FPN regions in relation to inattention scores in children, we conducted link-by-link *Pearson*'s correlations and multiple comparisons were corrected using FDR (p<0.05).

Brain state dynamics differentiate TD and ADHD children

To examine whether brain state dynamics could successfully differentiate TD children and children with ADHD, we conducted multivariate classification analysis using linear support vector machine (SVM). The occupancy rate and mean lifetimes on each latent brain state were

used as features to predict group identity of each child (TD or ADHD), separately. The model was evaluated using LOOCV. Each time, one data point was selected as a test set and the rest of the data were used as a training set. The training set was then used to train a SVM model, which was then applied to the test set for classification. This procedure was repeated N times with each data point used exactly once as a test set. The significance of classification accuracy was evaluated using permutation (500 times).

Sample size and power calculation

Since no study has investigated the relationship between latent brain state and behavioral variability in children with ADHD, the power calculation in the current study is based on previous meta-analysis focusing on IIRV difference between children with ADHD and TD children [9], suggesting a sample size of 22 subjects per group would have greater than 80% power to detect group difference in IIRV between children with ADHD and TD children.

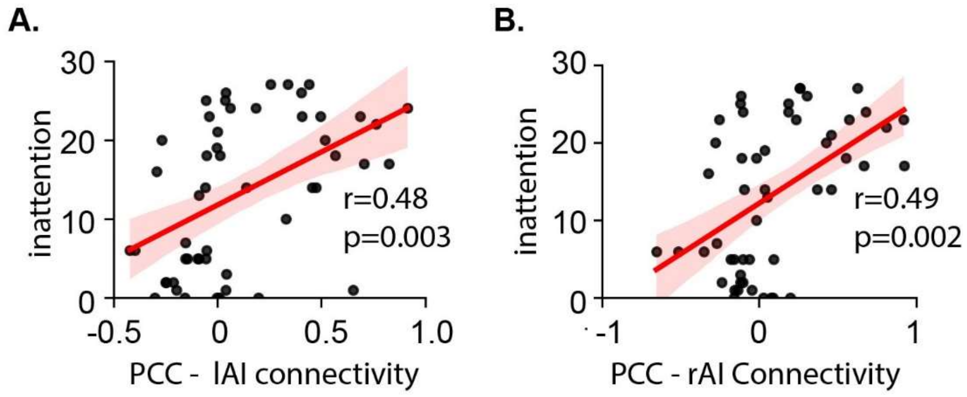

Figure S1. (A) Inattention scores were associated with functional connectivity between PCC and lAI, and **(B)** PCC and rAI in latent brain S1 (*ps*<0.05, FDR corrected).

Table S1 Summary of demographic information, clinical symptoms, head motion, and behavioral performance in TD children and children with ADHD.

	TD	ADHD	t/chi-stats	p-value
sample size	23	29		
age (years old)	10.5±1.2	10.7±1.2	0.62	0.5
gender (F/M)	11/12	11/18	0.19	0.6
Verbal IQ	112±14	107±14	1.32	0.19
Inattention	5±6	20±5	8.81	1.38E-11
Hyperactivity/Impulsivity	3±5	13±6	6.27	7.83E-08
Total displacement (mm)	1.9±1.1	2.2±1.0	0.93	0.3
Framewise displacement (mm)	0.06±0.03	0.07±0.02	1.33	0.2
Accuracy (%)	96±2	93±5	3.08	0.004
RT mean (ms)	467±50	485±59	1.15	0.2
RT std (ms)	88±27	110±35	2.57	0.01
RT mu (ms)	408±42	407±43	0.02	0.9
RT sigma (ms)	52±15	58±17	1.35	0.2
RT tau (ms)	60±29	77±34	2	0.05
HDDM a	1.8±0.4	1.9±0.6	1.27	0.2
HDDM v	3.6±0.9	2.9±0.9	2.95	0.005
HDDM t	0.22±0.07	0.17±0.09	2.7	0.01

FD: Frame-wise displacement

Table S2. Multiple linear regression analysis showed that occupancy rate (OR) of the latent state S1 is a robust predictor of IIRV, including RT Std and Tau.

	betas	*t value*	*p value*
Std~OR(S1)+Age+Gender+IQ+FD			
OR(S1)	-129.8	-2.79	0.008*
Age	-3.38	-0.93	0.36
Gender	-27.6	-3.34	0.002*
IQ	-0.07	-0.22	0.83
FD	45.83	0.29	0.78
Tau~OR(S1)+Age+Gender+IQ+FD			
OR(S1)	-118.6	-2.62	0.01*
Age	-2.96	-0.84	0.41
Gender	-30.33	-3.78	0.001*
IQ	-0.02	-0.08	0.9
FD	9.76	0.06	0.94
Std~OR(S2)+Age+Gender+IQ+FD			
OR(S2)	81.88	2.05	0.04*
Age	-2.24	-0.59	0.56
Gender	-29.04	-3.44	0.001*
IQ	-0.24	-0.8	0.45
FD	-32.78	-0.2	0.84
Tau~OR(S2)+Age+Gender+IQ+FD			
OR(S2)	84.65	2.2	0.03*
Age	-2.09	-0.57	0.58
Gender	-31.89	-3.92	0.0003*
IQ	-0.18	-0.61	0.55
FD	-71.96	-0.45	0.65

Table S3. Multiple linear regression analysis showed that occupancy rate (OR) of the latent state S1 is a robust predictor of information accumulation speed (v) during decision-making process.

	betas	*t value*	*p value*
v~OR(S1)+Age+Gender+IQ+FD			
OR(S1)	2.92	2.16	0.04*
Age	0.03	0.23	0.82
Gender	0.03	0.14	0.89
IQ	0.01	1.09	0.28
FD	-0.49	-0.11	0.92
v~OR(S2)+Age+Gender+IQ+FD			
OR(S2)	-2.01	-1.68	0.1
Age	0.02	0.26	0.8
Gender	0.07	0.28	0.77
IQ	0.01	1.13	0.27
FD	0.07	0.02	0.99

Table S4. Support vector regression analysis with cross-validation showed that occupancy rates and mean lifetimes of latent state S2, no other latent states, predicted inattention symptoms.

Features	Inattention		HyperImpul	
	Pearson's correlation	p-value	*Pearson*'s correlation	p-value
S1	0.06	0.68	0.05	0.71
S2	0.29	0.04*	0.05	0.72
S3	0.12	0.41	-0.07	0.61
S4	-0.73	2.00E-09	-0.09	0.51

Table S5. Classification analysis using linear support vector machine (C=1) showed that occupancy rates and mean lifetimes of latent state S2, no other latent states, distinguish children with ADHD from TD children.

Features	CV ACC (%)	p-value
S1	55	0.54
S2	65	0.03*
S3	56	0.62
S4	56	0.58

Reference

1. Ratcliff R, McKoon G. The diffusion decision model: theory and data for two-choice decision tasks. *Neural computation* 2008; **20**(4)**:** 873-922.

2. Wiecki TV, Sofer I, Frank MJ. HDDM: Hierarchical Bayesian estimation of the Drift-Diffusion Model in Python. *Frontiers in neuroinformatics* 2013; **7:** 14.

3. Taghia J, Cai WD, Ryali S, Kochalka J, Nicholas J, Chen TW *et al.* Uncovering hidden brain state dynamics that regulate performance and decision-making during cognition. *Nat Commun* 2018; **9**.

4. Bishop CM. *Pattern Recognition and Machine Learning*. Springer: New York, 2006.

5. Everitt BS. *An introduction to latent variable models*. Chapman & Hall: London, 1984.

6. Ghahramani Z, Beal MJ. Variational inference for Bayesian mixtures of factor analysers. *Adv Neur In* 2000; **12:** 449-455.

7. Fox E, Sudderth E, Jordan M, Willsky A. Nonparametric Bayesian learning of switching dynamical systems. *Advances in Neural Information Processing Systems* 2009; **21:** 457-464.

8. Friedman J, Hastie T, Tibshirani R. Regularization Paths for Generalized Linear Models via Coordinate Descent. *J Stat Softw* 2010; **33**(1)**:** 1-22.

9. Kofler MJ, Rapport MD, Sarver DE, Raiker JS, Orban SA, Friedman LM *et al.* Reaction time variability in ADHD: a meta-analytic review of 319 studies. *Clin Psychol Rev* 2013; **33**(6)**:** 795-811.
